# Blue Cone Monochromatism with Foveal Hypoplasia Caused by the Concomitant Effect of Variants in *OPN1LW/OPN1MW* and *GPR143* Genes

**DOI:** 10.3390/ijms22168617

**Published:** 2021-08-10

**Authors:** Giancarlo Iarossi, Andrea Maria Coppè, Chiara Passarelli, Paolo Enrico Maltese, Lorenzo Sinibaldi, Alessandro Cappelli, Sarah Cetola, Antonio Novelli, Luca Buzzonetti

**Affiliations:** 1 Department of Ophthalmology, Bambino Gesù Children’s Hospital, 00165 Rome, Italy; andreamaria.coppe@opbg.net (A.M.C.); alessandro.cappelli@opbg.net (A.C.); luca.buzzonetti@opbg.net (L.B.); 2 Translational Cytogenomics Research Unit, Bambino Gesù Children’s Hospital, IRCCS, 00146 Rome, Italy; chiara.passarelli@opbg.net (C.P.); lorenzo.sinibaldi@opbg.net (L.S.); sarah.cetola@opbg.net (S.C.); antonio.novelli@opbg.net (A.N.); 3 MAGI’S Lab s.r.l., 38068 Rovereto, Italy; 4 Rare Disease and Medical Genetics, Bambino Gesù Children’s Hospital, IRCCS, 00146 Rome, Italy

**Keywords:** blue cone monochromatism, X-linked inheritance, foveal hypoplasia, *OPN1LW/OPN1MW* gene cluster

## Abstract

Blue cone monochromatism (BCM) is an X-linked recessive cone dysfunction disorder caused by mutations in the *OPN1LW/OPN1MW* gene cluster, encoding long (L)- and middle (M)-wavelength-sensitive cone opsins. Here, we report on the unusual clinical presentation of BCM caused by a novel mutation in the *OPN1LW* gene in a young man. We describe in detail the phenotype of the proband, and the subclinical morpho-functional anomalies shown by his carrier mother. At a clinical level, the extensive functional evaluation demonstrated in the proband the M/L cone affection and the sparing of S-cone function, distinctive findings of BCM. Interestingly, spectral-domain optical coherence tomography showed the presence of foveal hypoplasia with focal irregularities of the ellipsoid layer in the foveal area, reported to be associated with some cases of cone-rod dystrophy and achromatopsia. At a molecular level, we identified the novel mutation c.427T > C p.(Ser143Pro) in the *OPN1LW* gene and the common missense mutation c.607T > C (p.Cys203Arg) in the *OPN1MW* gene. In addition, we discovered the c.768-2_769delAGTT splicing variant in the *GPR143* gene. To our knowledge, this is the first case of foveal hypoplasia in a BCM patient and of mild clinical affection in a female carrier caused by the concomitant effect of variants in *OPN1LW/OPN1MW* and *GPR143* genes, thus as the result of the simultaneous action of two independent genetic defects.

## 1. Introduction

Blue cone (S cone) monochromatism (BCM) is a rare X-linked congenital cone dysfunction syndrome, caused by mutations in the *OPN1LW*/*OPN1MW* gene cluster on the X chromosome. The cluster contains a single *OPN1LW* and one or more copies of the *OPN1MW* gene and controls the expression of the red (L, long wavelength) and green (M, middle wavelength) cone photoreceptor opsins. The genes expressing the opsin for the third cone subtype, S (short wavelength) or blue cones (*OPN1SW*), and the rod pigment are autosomal and not affected in BCM [1,2,3,4]. Consequently, vision in affected males is subserved only by S cone and rod function, as no functional L or M cones are present in the retina while tritan discrimination [5], which has been reported to deteriorate with increasing illuminance [6] and scotopic perception (i.e., night vision, subserved by rod function, to be distinguished from the photopic, or day vision, primarily due to cone function, which is also responsible for color perception) are retained. The prevalence is approximately 1 in 100,000 individuals. Color discrimination is severely impaired from birth and BCM patients typically present reduced visual acuity (6/24 to 6/60), pendular nystagmus, photophobia, and often have a myopic pattern.

BCM presents clinical and functional differences with achromatopsia (ACHM). ACHM is an autosomal recessive condition characterized by absent cone function and normal rod function while BCM has an X-linked inheritance pattern with a preserved tritan cone and scotopic function. The two conditions can be differentiated by psychophysical and electroretinogram (ERG) assessment, with BCM showing a profoundly reduced (but detectable) photopic ERG response with a preserved S cone ERG and ACHM showing an absent cone function. Moreover, patients affected by BCM often have myopia while ACHM is mainly associated with a hypermetropic refractive error. Despite both diseases having traditionally been considered stationary, recent reports have demonstrated evidence of slow mutation-dependent progression over time [7,8,9,10,11,12,13]. As fundus appearance can be normal in early stages in both conditions with variable retinal pigment epithelial alterations, the clinical diagnosis can be challenging in early infancy in a male patient until a detailed color vision or ERG testing can be performed. Consequently, genetic testing assumes a relevant role to clarify the diagnosis.

BCM is mainly caused by five categories of mutations affecting *OPN1LW* and *OPN1MW* loci: (i) a one-step pathway whereby the locus control region (LCR), a conserved sequence roughly 3.5 kb upstream of the L gene that regulates the expression of the L and M genes ensuring that only one opsin gene in the array is expressed in a single cone photoreceptor, is partially or completely deleted; (ii) a two-step mutation mechanism of non-homologous recombination and point mutation where the non-homologous recombination between the L and M opsin genes reduces the number of genes in the opsin array to one, followed by a subsequent point mutation (most commonly a missense variant), which inactivates the residual gene leading to a loss of functional L cones and M-cones; (iii) a third class of mutation where a single opsin array gene (L) was found to have a deletion of an entire exon (exon 4); (iv) a gene conversion transferring a mutation between *OPN1LW* and *OPN1MW*; (v) a category involving a combination of common variants in exon 3 in an otherwise normal gene array known as “exon 3 single-nucleotide polymorphism interchange haplotypes”, which result in aberrant splicing. Spectral-domain optical coherence tomography (SD-OCT) analysis of patients affected by BCM has shown a variable macular thinning associated with a wide spectrum of photoreceptor integrity and focal ellipsoid disruption in the area corresponding to the normal S cone-free zone. The identification of the residual cone structure confirmed by adaptive optics scanning light ophthalmoscopy (AOSLO) [4,14], and previous studies reporting a successful gene addition therapy in adult dichromatic monkeys lacking the L-opsin gene, resulting in a trichromatic visual behavior [15], and restoration of cone function in a rat model [16] have suggested BCM as a candidate disease for gene augmentation therapy.

In the present report, we identified a novel X-linked mutation in the *OPN1LW* gene in a young male patient and described in detail the unusual phenotype of the proband due to the concomitant effect of variants in the *OPN1LW*/*OPN1MW* and *GPR143* genes and the subclinical morpho-functional alterations of the mother, thus showing the result of the simultaneous action of two independent genetic defects.

## 2. Results

### 2.1. Clinical Report of the Proband and the Carrier

Proband was a male patient, aged 29 at the time of our first observation, born from non-consanguineous parents and not presenting apparent systemic abnormal features. Familiar history was unremarkable for relevant ocular disorders.

The patient reported photophobia, nystagmus, color vision impairment, and low vision since early childhood. No night vision difficulty was referred. His visual acuity was 1.00 LogMAR with a refractive error of −1.50 sph and −2.50 cyl/180 in RE and 1.00 LogMAR with a refractive error of −2.00 sph and −2.50 cyl/180 in LE.

**Figure 1:**
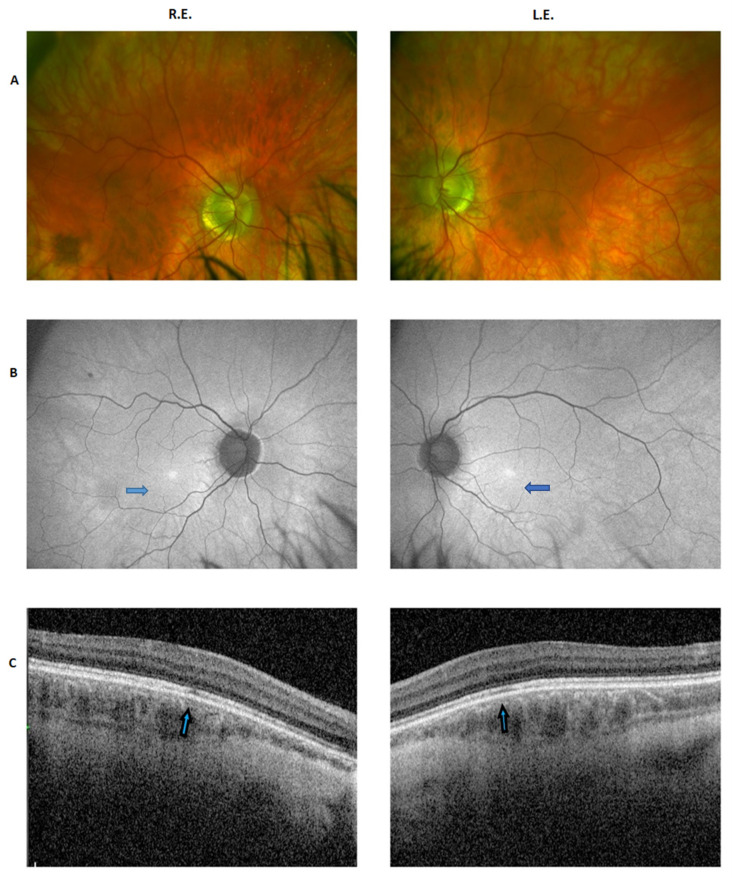
Ophthalmic features of the proband. (**A**) Fundus images (Daytona wide-field retinography) of the right and left eye showing a myopic pattern with relatively pale optic disk, regular vessel caliber, and mild RPE mottling. (**B**) FAF images (Daytona wide-field retinography) showing a slightly abnormal distribution of the superficial retinal vessels and a hyperfluorescent irregular elliptic area of approximatively 400 µm diameter corresponding to the hypoplasic fovea (arrows). (**C**) SD-OCT macular scan showing the absence of foveal depression, a thickening of the deep choroideal vessels (Haller’s layer), and a thinning and slight fragmentation of the ellipsoid layer in the central fovea (arrows) corresponding to an areola of approximately 300 µ diameter, associated with fragmentation of the corresponding external limiting membrane (EML). A slight widening of the outer nuclear layer (ONL) and inner nuclear layer (INL) in the same area is associated with irregular optical density of the INL.

Fundus examination showed a myopic pattern with relative pale optic disk, regular vessel caliber, and mild RPE mottling. SD-OCT showed the absence of foveal depression, a thickening of the deep choroideal vessels (Haller’s layer), and a thinning and slight fragmentation of the ellipsoid layer in the central fovea corresponding to an areola of approximately 300 µm diameter, associated with fragmentation of the corresponding external limiting membrane (EML). A slight widening of the outer nuclear layer (ONL) and inner nuclear layer (INL) in the same area is associated with irregular optical density of the INL. The FAF images showed a slightly abnormal distribution of the superficial retinal vessels and an hyperfluorescent irregular elliptic area of approximatively 400 µm diameter corresponding to the hypoplasic fovea (Figure 1).

**Figure 2:**
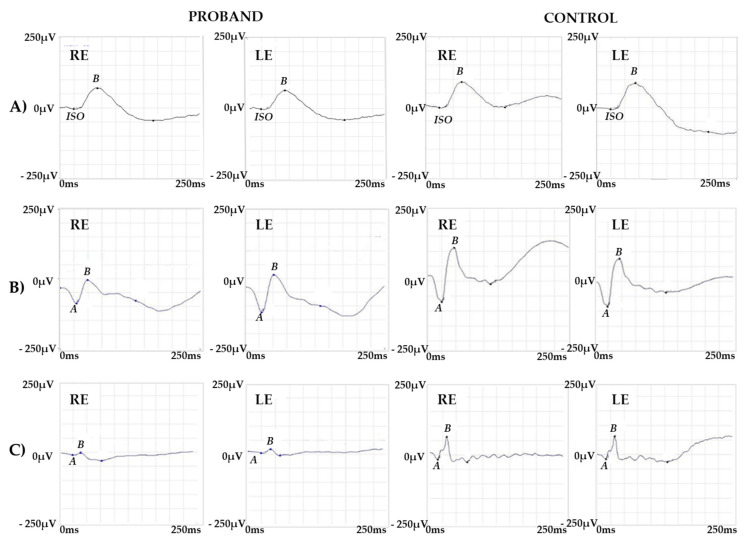
ERG Responses. ERG recordings showing (**A**) a normal amplitude response for the scotopic stimulus, (**B**) a slightly reduced response for the combined stimulus and (**C**) a severely reduced response for the photopic stimulus. Representative examples of ERG responses from a normal subject are shown for comparison. Legend: RE, right eye; LE, left eye; ISO, isoelectric line; A, a-wave; B, b-wave.

The Goldmann manual kinetic visual field was normal in both eyes. Red–green color vision defect was found in the Panel D15 and Ishihara tests. A protan ordering of the Panel D15 was displayed, as previously described in BCM [17]. ERG recordings showed a normal scotopic response, a slightly reduced combined response, and severely reduced but detectable photopic response (Figure 2).

**Figure 3:**
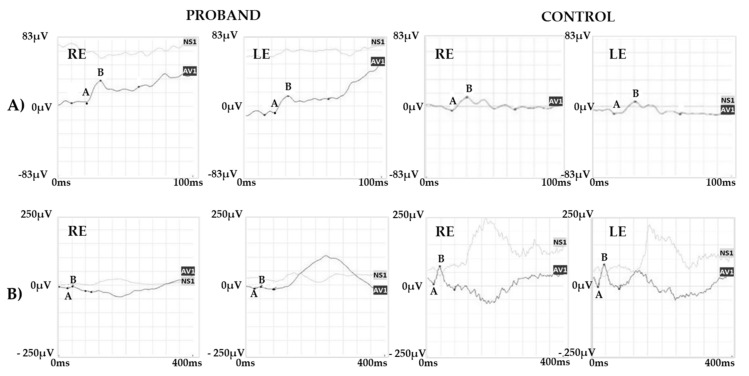
Specialized ERG responses. (**A**) S cone and (**B**) M-L cone-mediated ERG recordings from the proband and a control subject. Note that S cone ERG shows a normal amplitude response while the M-L cone response is delayed and severely reduced but still recordable as compared with the normal control. Legend: RE, right eye; LE, left eye; AV1, average response; NS1, noise signal; A, a-wave; B, b-wave.

Specialized ERG recorded using chromatic stimuli showed a severely reduced but still recordable and delayed M-L-mediated response and a normal S cone-mediated response. In normal individuals, the ML cone ERG is three times larger in amplitude (a-b wave peak) and 10 ms shorter (b-wave peak) compared to S cone ERG. In this particular patient, S cone ERG presented a simplified waveform and larger amplitude response as compared to the ML cone response (Figure 3).

The patient’s mother, carrying the same mutations, reported a subnormal visual acuity since childhood without other relevant symptoms. Her visual acuity was 0.09 LogMAR in both eyes with a refractive error of −2.00 sph and in RE and −1.50 sph in LE.

**Figure 4:**
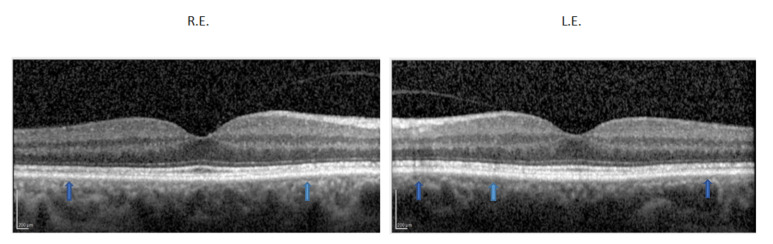
SD-OCT macular scan of the carrier showing a preserved foveal morphology with mild diffuse irregularity of the ellipsoid and retinal pigmented epithelium layers (arrows) in both eyes.

Optic media were clear and fundus examination was apparently normal for age. SD-OCT showed a preserved foveal morphology with mild diffuse irregularity of the ellipsoid and retinal pigmented epithelium layers in both eyes (Figure 4).

**Figure 5:**
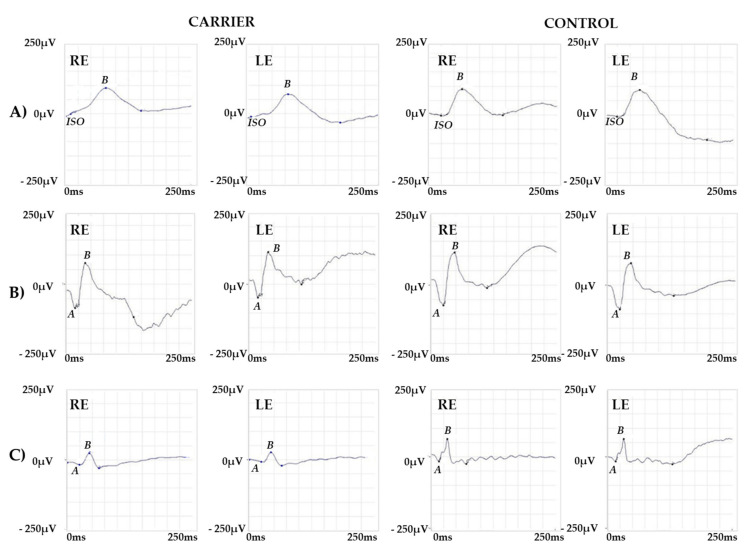
ERG Responses. ERG recordings showing (**A**) a normal scotopic and (**B**) combined response and (**C**) a reduced photopic response. Representative examples of ERG responses from a normal subject are shown for comparison. Legend: RE, right eye; LE, left eye; ISO, isoelectric line; A, a-wave; B b-wave.

Color vision evaluated with Panel D15 and Ishihara test showed a mild defect in the red/green axis. ERG recordings showed a normal scotopic and combined response and a reduced photopic response (Figure 5).

**Figure 6:**
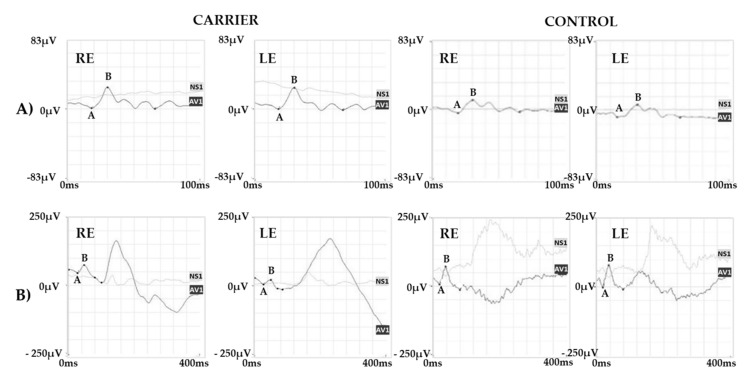
Specialized ERG responses. (**A**) S cone and (**B**) M-L cone-mediated ERG recordings from the carrier and a control subject. Note that S cone ERG shows a normal amplitude response while the M-L cone response is delayed and reduced as compared with the normal control. Legend: RE, right eye; LE, left eye; AV1, average response; NS1, noise signal; A, a-wave; B, b-wave.

Specialized ERG recorded using chromatic stimuli showed a slightly reduced and delayed M-L-mediated response and a normal S cone-mediated response (Figure 6).

### 2.2. Genetic Analysis

Genetic testing revealed a novel hemizygous variant c.427T > C, p.(Ser143Pro) in the *OPN1LW* gene that was confirmed by a family segregation study. The variant has been classified by American College of Medical Genetics and Genomics (ACMG) guidelines [18], with the help of the online software VarSome (https://varsome.com/ accessed on 4 May 2021) as a variant of unknown significance (VUS) according to these scores: PM2, variant not found in gnomAD exomes; PP3, pathogenic computational verdict based on 9 pathogenic predictions from BayesDel_addAF, DANN, FATHMM-MKL, LIST-S2, M-CAP, MVP, MutationTaster, PrimateAI, and SIFT vs. no benign predictions. The patient also presented the common missense mutation c.607T > C (p.Cys203Arg) in the *OPN1MW* gene, an already described variant causing deutan color vision deficiency when present in the M gene [19], and the c.768-2_769delAGTT splicing variant in the *GPR143* gene, known as being related to ocular albinism type I (OMIM # 300500) and X-linked congenital nystagmus-6 (OMIM # 300814) [20], both in hemizygous status and inherited from the mother.

## 3. Discussion

BCM or X-linked incomplete ACHM [21] has been matter of clinical, electrophysiological, and psychophysical investigation for a long time [22,23] and more recent studies on the molecular genetic basis of cone opsin deficiencies [3,4,5,6,7,8,9,10,11,12,13,14,15,16,17,18,19,20,21,22,23,24,25] have led to a better characterization of the molecular mechanisms causing the disease. The described evidence of a sufficient M/L cone sparing with preserved photoreceptor lamination at the fovea in humans [4,26] and proof of dissociation of structure and function suggested BCM as a candidate for gene augmentation therapy. The successful treatment of color blindness in adult monkeys [15], the restoration of cone function in a rat model [16], and other deeper studies on the function of remaining photoreceptor has confirmed the potential effectiveness of gene therapy in BCM [27]. Despite the different molecular genetic mechanisms responsible for the disease, the phenotypes so far reported were relatively homogeneous and mostly characterized by a normal myopic retina with macular retinal pigment epithelial disturbance and atrophy in older patients and significant macular thinning associated with ellipsoid layer disruption at the SD-OCT analysis.

**Figure 7:**
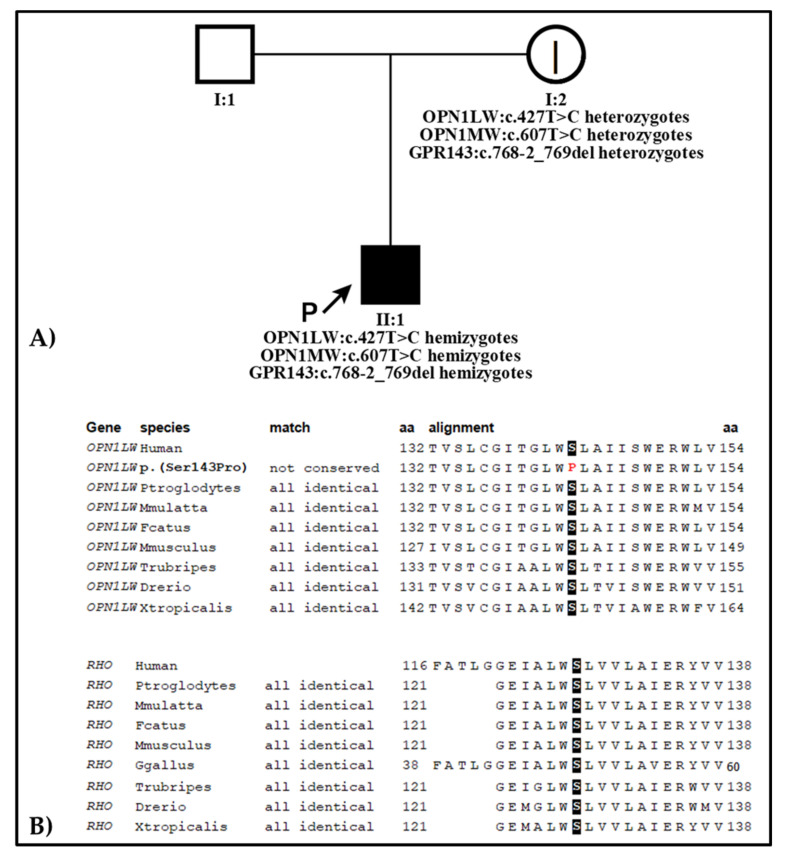
(**A**) Family tree showing the X-linked recessive transmission of the two diseases. Legend: P, proband; |, subclinic phenotype; (**B**) Amino acid (aa) conservation across species of the novel *OPN1LW* variant showing that long-wave-sensitive opsin-1 Ser143 is highly conserved. Ser143 in the *OPN1LW* gene corresponds to Ser127 in the human rhodopsin gene *RHO*; the alignment shows that the amino acid serine is conserved across species also in *RHO;* this variant was not found in any queried database and was classified as likely pathogenic by VarSome, thus further supporting the evolutionary and functional importance of the residue.

In our report, we described an unusual clinical presentation of BCM with foveal hypoplasia caused by a novel mutation in the *OPN1LW* gene and the concomitant effect of variants in the *OPN1MW* and *GPR143* genes. The proband, affected since early childhood by the typical clinical signs commonly associated with a severe cone dystrophy, has been only recently diagnosed as having BCM after genetic testing and a detailed morpho-functional study, which led to a proper phenotype/genotype correlation. Indeed, though the novel mutation of the *OPN1LW* gene identified in this patient has been classified as a VUS, its disease causative role can be sustained by the following findings: the variant was not found in any queried public database; it is in a hemizygous state, inherited by the mother showing mild clinical sign of the disease and determining a full phenotype only in the proband in line with the X-linked transmission of the disease; it affects a highly conserved amino acid of the protein across species (Figure 7); and the presence of the common missense mutation c.607T > C (p.Cys203Arg) in the *OPN1MW* gene.

Moreover, the extensive functional evaluation, carried out with psychophysical and electrophysiological testing, demonstrated the M/L cone affection and the sparing of S cone function, typical findings in BCM patients. Differently from the common morphological presentation of BCM, SD-OCT showed the presence of foveal hypoplasia (FH) associated with focal irregularities and fragmentation of the ellipsoid layer in the foveal area. Foveal hypoplasia has been divided into a typical and an atypical form [28]. Typical FH has been described in albinism, *PAX6* mutations, *SLC38A8* mutations, retinopathy of prematurity, optic nerve hypoplasia, and isolated cases of arrested retinal development in different disorders, and is characterized by the continuation of inner retinal layers posterior to the foveola. The atypical form can present an additional disruption of the outer retinal layers or abnormal lamination pattern and has been associated with ACHM and cone-rod dystrophy. To our best knowledge, this is the first reported case of BCM caused by mutations in the *OPN1LW/OPN1MW* genes associated with FH due to the concomitant effect of the *GPR143* gene. GPR143, a transmembrane receptor in melanocytes, controls several RPE activities that are likely to play an important role in retinal development and could contribute to retinal health and disease [29]. As previously reported, the *GPR143* gene has been associated with ocular albinism, an ophthalmic disorder typically presenting foveal hypoplasia, congenital nystagmus, fundus hypopigmentation, and a variable grade of iris transluminance. Interestingly, our patient showed neither systemic nor some of the overmentioned ocular features, such as fundus depigmentation or iris transilluminance. However, this is in accordance with a previous paper reporting a less severe effect on pigmentation of *GPR143* as compared to other genes involved in the pathogenesis of ocular albinism [20]. Based on the timing of developmental arrest, typical FH has been classified into four grades with an additional grade for atypical FH. In our case report, the proband showed a foveal pattern consistent with grade three of the grading scale of typical FH consisting in absent extrusion of the plexiform layer, absence of a foveal pit, and outer nuclear layer widening and a focal foveal area of photoreceptor disruption reported in atypical FH. This presentation is consistent with an early arrest in the process of foveal differentiation and with the severe form of congenital nystagmus and poor vision of the patient [30]. Differently from some forms of ACHM where a substantial disruption of the photoreceptor layer can be observed [31], in our patient, only focal irregularities and fragmentation of the ellipsoid layer were present. Though we cannot discriminate the combined or selective effect of the mutations found or exclude unknown additional factors that affected the foveal development of the patient, this finding is consistent with the SD-OCT reports on BCM patients describing a variable grade of photoreceptor alteration. Moreover, it must be considered that, despite using a SD-OCT with a fast scanning speed of 85,000 Hz, the acquisition and analysis quality might have been affected by the severe nystagmus, limiting a deeper analysis of the photoreceptor layer.

The family segregation study confirmed the causative role of the *OPN1LW* mutation by the carrier status of the proband’s mother. The morpho-functional analysis of the carrier phenotype showed a subtle alteration of the outer retinal layers associated with a reduced photopic ERG due to the dysfunction of the L/M-mediated response. Previous reports showed ERG and eye movement abnormalities in BCM carriers without describing in detail the retinal morphologic alteration [32,33]. A variable extent of abnormalities has been described in carriers of X-linked inherited retinal dystrophies. Carrier females generally retain good retinal function despite the presence of focal areas of retinal degeneration, which are probably due to random X inactivation. The severity of retinal disease in carriers is variable and, therefore, depends on the proportion of cells expressing the mutant X chromosome. Though widely reported in other more common X-linked retinal dystrophies, such as retinitis pigmentosa or choroideremia, to our knowledge, this is the first report describing the morpho-functional alterations in a BCM carrier, strengthening the causative role of the novel variant identified in the *OPN1LW* gene.

In conclusion, in this report, we have described the first case of foveal hypoplasia in BCM patients and evidence of mild clinical affection in a carrier caused by a novel variant in the *OPN1LW* gene and the concomitant effect of variants in the *OPN1MW* and *GPR143* genes, thus, presenting the result of the simultaneous action of two independent genetic defects.

## 4. Materials and Methods

### 4.1. Patient Studies and Clinical and Ophthalmological Examinations

All procedures in this study adhered to the tenets of the Declaration of Helsinki and were approved by the Ethic Committee of the Bambino Gesù Children’s Hospital (Code 558/2012). Appropriate informed consent was obtained from the patient and the parents. The proband and his family underwent comprehensive age-appropriate ophthalmic examination, including best corrected visual acuity (BCVA) measurement with the Early Treatment Diabetic Retinopathy Study (ETDRS) charts, expressed as a logarithm of the minimum angle of resolution (logMAR); slit-lamp biomicroscopy; indirect ophthalmoscopy with 15D noncontact lens (Volk); color fundus photos and fundus autofluorescence (FAF; Daytona wide-field retinography, Optos, Marlborough, MA, USA); spectral-domain optical coherence tomography (SD-OCT) (Heidelberg Spectralis OCT2, Heidelberg Engineering, Heidelberg, Germany); and full-field ERG recording according to the ISCEV standards (Retimax recording system, CSO, Florence, Italy). In addition, we recorded the S cone ERG, as previously described [34], using chromatic stimuli to obtain a combined waveform, in which the S cone response (S cone b-wave) follows the L cone and M cone response (L-M cone b-wave). Briefly, S cone-mediated ERGs were recorded in response to a blue (420 nm) Ganzfeld stimulus presented on a yellow background. ML cone-mediated ERGs were obtained in response to a red (580 nm) stimulus on a green background.

### 4.2. Genetic Testing

Genetic testing was performed at the genetic laboratories of the Bambino Gesù Children’s hospital. DNA was extracted from peripheral blood with Qiagen columns (QIAamp DNA minikit; Qiagen, Hilden, Germany) according to the manufacturer’s instructions. The concentration and purity of DNA samples were quantified by an ND-1000 spectrophotometer (NanoDrop; Thermo Scientific, Waltham, MA, USA) and by a FLx800 Fluorescence Reader (BioTek, Winooski, VT, USA). A customized panel sequencing was performed on genomic DNA by using the kit Twist Custom Panel (Twist Bioscience, South San Francisco, CA, USA) according to the manufacturer’s protocol on a NovaSeq6000 platform (Illumina, San Diego, CA, USA). The reads were aligned to human genome build GRCh37/UCSC hg19. The Dragen Enrichment application of BaseSpace (Illumina) and TGex software (LifeMap Sciences, Inc., Alameda, CA, USA) were used for the variant calling and annotating variants, respectively. Sequence data were carefully analyzed, and the presence of all suspected variants was checked in the public databases (dbSNP, Exome Aggregation Consortium (ExAC) https://www.ncbi.nlm.nih.gov/snp/ accessed on 5 May 2021) and Genome Aggregation Database (gnomAD https://gnomad.broadinstitute.org/ accessed on 5 May 2021)). The variants were evaluated by VarSome [35] and categorized in accordance with the ACMG recommendations [18]. Variants were examined for coverage and Qscore (minimum threshold of 30) and visualized by the Integrative Genome Viewer (IGV). The *OPN1LW* (NM_020061.5) and *OPN1MW* (NM_000513.2) gene cluster is generally located head-to-tail in a tandem arrangement with a single *OPN1LW* followed by one or more *OPN1MW* copies (the copy number is not detectable by our sequencing analysis). The high degree of sequence homology made the analysis difficult, especially for the *OPN1MW* gene. The *OPN1LW, OPN1MW*, and *GPR143* (NM_000273.3) variants were confirmed in the patient and his mother by Sanger sequencing, the formers by a long-range PCR protocol as previously reported by Katagiri et al. [19].

## Data Availability

Data is contained within the article.

## References

[1] Nathans J., Thomas D., Hogness D. (1986). Molecular genetics of human color vision: The genes encoding blue, green, and red pigments. Science.

[2] Nathans J., Davenport C., Maumenee I., Lewis R., Hejtmancik J., Litt M., Lovrien E., Weleber R., Bachynski B., Zwas F. (1989). Molecular genetics of human blue cone monochromacy. Science.

[3] Neitz J., Neitz M. (2011). The genetics of normal and defective color vision. Vis. Res..

[4] Cideciyan A.V., Hufnagel R.B., Carroll J., Sumaroka A., Luo X., Schwartz S.B., Dubra A., Land M., Michaelides M., Gardner J.C. (2013). Human Cone Visual Pigment Deletions Spare Sufficient Photoreceptors to Warrant Gene Therapy. Hum. Gene Ther..

[5] Gardner J.C., Michaelides M., Holder G.E., Kanuga N., Webb T., Mollon J., Moore A.T., Hardcastle A.J. (2009). Blue cone monochromacy: Causative mutations and associated phenotypes. Mol. Vis..

[6] Young R.S., Price J. (1985). Wavelength discrimination deteriorates with illumination in blue cone monochromats. Investig. Ophthalmol. Vis. Sci..

[7] Michaelides M., Johnson S., Simunovic M., Bradshaw K., Holder G., Mollon J., Moore A.T., Hunt D.M. (2004). Blue cone monochromatism: A phenotype and genotype assessment with evidence of progressive loss of cone function in older individuals. Eye.

[8] Khan N.W., Wissinger B., Kohl S., Sieving P.A. (2007). CNGB3Achromatopsia with Progressive Loss of Residual Cone Function and Impaired Rod-Mediated Function. Investig. Opthalmol. Vis. Sci..

[9] Thiadens A.A.H.J., Somervuo V., Born L.I.V.D., Roosing S., van Schooneveld M.J., Kuijpers R.W.A.M., van Moll-Ramirez N., Cremers F.P.M., Hoyng C.B., Klaver C.C.W. (2010). Progressive Loss of Cones in Achromatopsia: An Imaging Study Using Spectral-Domain Optical Coherence Tomography. Investig. Opthalmol. Vis. Sci..

[10] Thomas M., McLean R.J., Kohl S., Sheth V., Gottlob I. (2012). Early signs of longitudinal progressive cone photoreceptor degeneration in achromatopsia. Br. J. Ophthalmol..

[11] Fahim A., Khan N.W., Zahid S., Schachar I.H., Branham K., Kohl S., Wissinger B., Elner V.M., Heckenlively J.R., Jayasundera T. (2013). Diagnostic Fundus Autofluorescence Patterns in Achromatopsia. Am. J. Ophthalmol..

[12] Aboshiha J., Dubis A.M., Cowing J.A., Fahy R.T.A., Sundaram V., Bainbridge J., Ali R., Dubra A., Nardini M., Webster A.R. (2014). A Prospective Longitudinal Study of Retinal Structure and Function in Achromatopsia. Investig. Opthalmol. Vis. Sci..

[13] Brunetti-Pierri R., Karali M., Melillo P., Di Iorio V., De Benedictis A., Iaccarino G., Testa F., Banfi S., Simonelli F. (2021). Clinical and Molecular Characterization of Achromatopsia Patients: A Longitudinal Study. Int. J. Mol. Sci..

[14] Carroll J., Dubra A., Gardner J.C., Mizrahi-Meissonnier L., Cooper R.F., Dubis A.M., Nordgren R., Genead M., Connor T.B., Stepien K.E. (2012). The Effect of Cone Opsin Mutations on Retinal Structure and the Integrity of the Photoreceptor Mosaic. Investig. Opthalmol. Vis. Sci..

[15] Mancuso K., Hauswirth W., Li Q., Connor T.B., Kuchenbecker J.A., Mauck M.C., Neitz J., Neitz M. (2009). Gene therapy for red–green colour blindness in adult primates. Nature.

[16] Zhang Z., Pang J., Xia F., Guo Q., Li L., An J., Zhang L., Hauswirth W.W., Yang S., Li Z. (2011). AAV-mediated gene therapy restores cone function in a rat with an M-cone Opsin deficiency, a model for blue cone monochromacy. Investig. Opthalmol. Vis. Sci..

[17] Weiss A.H., Biersdorf W.R. (1989). Blue cone monochromatism. J. Pediatr. Ophthalmol. Strabismus.

[18] Richards S., Aziz N., Bale S., Bick D., Das S., Gastier-Foster J., Grody W.W., Hegde M., Lyon E., Spector E. (2015). Standards and guidelines for the interpretation of sequence variants: A joint consensus recommendation of the American College of Medical Genetics and Genomics and the Association for Molecular Pathology. Genet. Med..

[19] Katagiri S., Iwasa M., Hayashi T., Hosono K., Yamashita T., Kuniyoshi K., Ueno S., Kondo M., Ueyama H., Ogita H. (2018). Genotype determination of the OPN1LW/OPN1MW genes: Novel disease-causing mechanisms in Japanese patients with blue cone monochromacy. Sci. Rep..

[20] Preising M.N., Forster H., Gonser M., Lorenz B. (2011). Screening of TYR, OCA2, GPR143, and MC1R in patients with congenital nystagmus, macular hypoplasia, and fundus hypopigmentation indicating albinism. Mol. Vis..

[21] Hess R.F., Mullen K.T., Sharpe L.T., Zrenner E. (1989). The photoreceptors in atypical achromatopsia. J. Physiol..

[22] Blackwell H., Blackwell O. (1961). Rod and cone receptor mechanisms in typical and atypical congenital achromatopsia. Vis. Res..

[23] Berson E.L., Sandberg M.A., Rosner B., Sullivan P.L. (1983). Color Plates to Help Identify Patients with Blue Cone Monochromatism. Am. J. Ophthalmol..

[24] Mizrahi-Meissonnier L., Merin S., Banin E., Sharon O. (2010). Variable Retinal Phenotypes Caused by Mutations in the X-Linked Photopigment Gene Array. Investig. Opthalmol. Vis. Sci..

[25] Nathans J., Maumenee I.H., Zrenner E., Sadowski B., Sharpe L.T., Lewis R.A., Hansen E., Rosenberg T., Schwartz M., Heckenlively J.R. (1993). Genetic heterogeneity among blue-cone monochromats. Am. J. Hum. Genet..

[26] Sumaroka A., Garafalo A.V., Cideciyan A.V., Charng J., Roman A.J., Choi W., Saxena S., Aksianiuk V., Kohl S., Wissinger B. (2018). Blue Cone Monochromacy Caused by the C203R Missense Mutation or Large Deletion Mutations. Investig. Opthalmol. Vis. Sci..

[27] Garafalo A.V., Cidecyan A.V., Hèon E., Sheplock R., Pearson A., Yu C.W., Sumaroka A., Aguirre G.D., Jacobson S.G. (2020). Progress in treating inherited retinal diseases: Early subretinal gene therapy clinical trials and candidates for future initiatives. Prog. Retin. Eye Res..

[28] Thomas M.G., Papageorgiou E., Kuht H.J., Gottlob I. (2020). Normal and abnormal foveal development. Br. J. Ophthalmol..

[29] McKay B.S. (2019). Pigmentation and Vision: Is GPR143 in Control?. J. Neurosci. Res..

[30] Rufai S.R., Thomas M.G., Purohit R., Bunce C., Lee H., Proudlock F.A., Gottlob I. (2020). Can Structural Grading of Foveal Hypoplasia Predict Future Vision in Infantile Nystagmus: A Longitudinal Study. Ophthalmology.

[31] Mastey R.R., Georgiou M., Langlo C.S., Kalitzeos A., Patterson E.J., Kane T., Singh N., Vincent A., Moore A.T., Tsang S.H. (2019). Characterization of Retinal Structure in ATF6-Associated Achromatopsia. Investig. Opthalmol. Vis. Sci..

[32] Berson E.L., Sandberg M.A., Maguire A., Bromley W.C., Roderick T.H. (1986). Electroretinograms in Carriers of Blue Cone Monochromatism. Am. J. Ophthalmol..

[33] Gottlob I. (1994). Eye movement abnormalities in carriers of blue-cone monochromatism. Investig. Ophthalmol. Vis. Sci..

[34] Sustar M., Hawlina M., Brecelj J. (2011). Electroretinographic evaluation of the retinal S-cone system. Doc. Ophthalmol..

[35] Kopanos C., Tsiolkas V., Kouris A., Chapple C.E., Aguilera M.A., Meyer R., Massouras A. (2018). VarSome: The human genomic variant search engine. Bioinformatics.

